# Genome-Wide Identification and Expression Analysis of the MADS Gene Family in Tulips (*Tulipa gesneriana*)

**DOI:** 10.3390/genes14101974

**Published:** 2023-10-22

**Authors:** Jiaojiao Lu, Lianwei Qu, Guimei Xing, Zhenlei Liu, Xiaochun Lu, Xiaori Han

**Affiliations:** 1 Liaoning Academy of Agriculture Sciences, Shenyang 110161, China; bestlj@163.com (J.L.); qulianwei2023@163.com (L.Q.); xingguimei0105@163.com (G.X.); liuzhenlei2023@163.com (Z.L.); 2 College of Land and Environment, Shenyang Agricultural University, Shenyang 110866, China

**Keywords:** MADS, gene expression, cold response, phylogeny, tulip

## Abstract

To investigate the cold response mechanism and low temperature regulation of flowering in tulips, this study identified 32 MADS-box transcription factor family members in tulips based on full-length transcriptome sequencing, named TgMADS1-TgMADS32. Phylogenetic analysis revealed that these genes can be divided into two classes: type I and type II. Structural analysis showed that *TgMADS* genes from different subfamilies have a similar distribution of conserved motifs. Quantitative real-time PCR results demonstrated that some *TgMADS* genes (e.g., *TgMADS3*, *TgMADS15*, *TgMADS16*, and *TgMADS19*) were significantly upregulated in buds and stems under cold conditions, implying their potential involvement in the cold response of tulips. In summary, this study systematically identified *MADS* family members in tulips and elucidated their evolutionary relationships, gene structures, and cold-responsive expression patterns, laying the foundation for further elucidating the roles of these transcription factors in flowering and the cold adaptability of tulips.

## 1. Introduction

Tulips require a period of low-temperature treatment for proper growth and flowering; however, the underlying molecular mechanism is still elusive. *MADS-box* genes are likely involved in the tulip’s response to cold treatment and the regulation of flowering [1,2]. The *MADS-box* gene family, as a wide range of transcription factor coding genes, is involved in the coding of transcription factors in almost all eukaryotes [3]. These transcription factors play an important role in development control and signal transduction in eukaryotes, especially in plants [4,5,6]. The *MADS-box* gene family is named from four subgene families: the *MCM1* (*MiniChromosome Maintenance 1*) gene in yeast, *AG* (*Agamous*) gene in *Arabidopsis thaliana*, *DEF* (*Deficiens*) gene in *Antirrhinum majus*, and human *SRF* (*Serum Response Factor*) gene [4,5]. They encode transcription factors with a common DNA binding domain MADS-box and recognize similar target DNA sequences [4]. In the following study, the *MADS-box* gene family is divided into two categories according to the presence of additional domains: type I and type II. Type I MADS contains only the SRF domain, while type II MADS contains a mediating domain (I-box) and a keratin helix domain (K-box) in addition to the MADS domain [7]. Since the type II *MADS-box* gene has a MADS-box domain at the N-terminus of the protein and an I-box and K-box at the C-terminus of the protein, the type II *MADS-box* gene is also called the MIKC type. With further research, according to the structural differences of the I-box domain, MIKC can be divided into two subgroups: MIKCC and MIKC* [8]. 

In plants, MADS-box proteins contain a DNA binding domain (M), mediating domain (I), keratin helix domain (K), and C-terminal domain (C), belonging to the MIKC type [3]. Members of the *MADS-box* gene family play an important role in many biological functions of plants. The transcription factors encoded by *MADS-box* genes control a variety of developmental processes from roots to flowers and fruits in flowering plants [9], such as biotic and abiotic stress tolerance, the flowering process, seed development, and fruit ripening [10]. Up to now, genome-wide identification methods have been carried out in various plants, including *Citrullus lanatus* [11], *Malus pumila* [12], *Brassica oleracea* [13], *Triticum aestivum* [14], *Zea mays* [15], *Gossypium* [8], *Chrysanthemum × morifolium* [16], *Cymbidium* [17], etc. The expression of related genes was also analyzed in *Tulipa gesneriana* [18,19,20], and the members of the *MADS-box* gene family in different plants were identified, and their functions were verified. The results of these studies have confirmed that the *MADS-box* gene family plays an important role in the homologous selection of floral organs, flowering time, and fruit ripening during plant growth and development [21,22].

In some flowering plants, mutations in *MADS-box* genes can lead to homologous transformation between different floral organs, which proves that these genes play a role in floral organ homology selection during development, and the genetic floral organ model is derived based on this phenomenon. The genetic floral organ model was developed from the ABC model proposed in 1991 [23] to a more accurate ABCDE model; that is, the MIKCC family genome merger determines the identity of floral organs: sepals (A + E), petals (A + B + E), stamens (B + C + E), carpels (C + E), and ovules (D + E) [24,25]. In previous studies, 39 *MIKCC* genes were found in *A. thaliana* [26]. 

It is worth mentioning that compared with higher dicotyledonous plants, there are many non-gramineous monocotyledonous plants, such as lilies and tulips. The floral organs of these plants are composed of three outer tepals, three inner tepals, six stamens, and three carpels. It is difficult to fully explain the morphology of this type of flower with the classical ABC model. In order to reveal the reasons for the formation of this flower morphology, researchers proposed a modified ABC model in 1993 [27], which was derived based on the morphological characteristics of wild-type and mutant tulip flowers. 

In addition to providing homologous and heterogeneous functions of plant flowers, members of the *MADS-box* gene family also affect the gene network of the reproductive development of flowering plants. Some *MADS-box* genes are flowering time genes, which depend on internal or environmental factors such as plant age, day length, and coldness to inhibit or promote flower transition [28,29]. 

Members of the *MADS-box* gene family are also involved in the development of seeds and fruits after flower fertilization. Studies have shown that some genes encode proteins required for the development of Arabidopsis fruit dehiscence, and there are also some genes encoding proteins required for the normal pattern of cell division, growth, and differentiation during the morphogenesis of long siliques [30]. 

In addition, studies have verified that in flowering plants, in addition to flowers and fruits, other tissues and organs such as embryos, roots, or leaves [31] also observed a large number of *MADS-box* gene family member expressions. Other studies have found the existence of *MADS-box* genes in gymnosperms, ferns, and even mosses. The above results indicate that the function of *MADS-box* gene family members is not limited to the development of flowers or fruits [32,33,34]. 

In summary, the *MADS* gene family is widely involved in the growth and development of various plants, and its importance is particularly reflected in the flowering process of plants. An in-depth understanding of *MADS* genes is essential for exploring the regulation of plant growth and development. However, as a very important flowering ornamental crop in the world, the research on the *MADS* gene family in tulips is very insufficient. On this basis, 32 *TgMADS* genes found in tulips were identified and divided into two major types and 15 subfamilies according to their domains. In addition, the expression patterns of all members under different treatments at room temperature and low temperature were also analyzed in this study, aiming to provide a basis for breeding cold tolerance in tulips, the molecular mechanism of low-temperature regulation of tulip flowering, and the functions of *MADS* genes. 

## 2. Materials and Methods

### 2.1. Plant Materials and Growth Conditions

The bulbs of tulip variety Dow Jones were preserved in our laboratory. The bulbs were first pretreated at 5 °C for four weeks. After that, the bulbs were treated at a low temperature: the temperature of the incubator was set to 5 °C, the treatment time points were 0 d, 15 d, 30 d, and 60 d, respectively, and the bulbs and buds were collected. In addition, the bulbs of different treatments were sown in nutrient soil with a photoperiod of 16/8 h (day/night) and a temperature of 5 °C. The bulbs were cultured for two weeks under conventional watering and fertilization conditions, and the stems of tulip plants were collected. After sampling, the samples were immediately placed in liquid nitrogen for quick freezing and then stored in a −80 °C refrigerator for subsequent research.

### 2.2. Identification and Sequence Analysis of TgMADS Gene Family Members

The reference sequence for the identification of the *TgMADS* gene family was derived from the full-length transcriptome of tulips obtained by previous sequencing in our laboratory. A total of 108 Arabidopsis MADS protein sequences were downloaded from the Arabidopsis Information Resource (TAIR, http://www.arabidopsis.org, accessed on 10 July 2023). According to the 108 AtMADS protein sequences, HMMER 3.0 software was used to construct the MADS protein Hidden Markov Model (HMM), and the potential TgMADS protein sequences were searched in all protein sequences of tulips according to the constructed HMM file [35]. For the accuracy of the results, blastp software (version: ncbi-blast-v2.10.1+) was used to compare the sequences of all proteins of tulips with the obtained TgMADS family reference sequences. The e-value was set to 10^−5^, and the sequences on the alignment were used as all potential TgMADS family sequences [36]. Finally, all the potential sequences obtained by the above two methods were used as candidate TgMADS family protein sequences, and the target sequences were subjected to domain annotation using the software PfamScan [37,38] (version: v1.6) and PfamA [39] (version: v33.1) databases. The sequence containing the PF00931 domain was used as the final TgMADS protein sequence. The subcellular localization of tulip TgMADS family members was predicted using the softberry online tool (http://linux1.softberry.com/berry.phtml?topic=index&group=programs&subgroup=proloc, accessed on 11 July 2023). The TgMADS protein was predicted by DeepTMHMM software (version 1.0.8) based on a deep learning model to determine whether it was a membrane protein [40]. In order to predict whether there is a potential signal peptide cleavage site in TgMADS protein and where it is located, this study used SignalP software (version v5.0b) to predict the protein based on a variety of artificial neural network algorithms [41].

### 2.3. Phylogenetic and Multiple Alignment Analysis of TgMADS Proteins

Multiple-sequence alignment of the identified MADS protein family sequences of *T. gesneriana* and *A. thaliana* was performed using mafft (v7.427) software [42], and then an NJ tree was constructed using MEGA10 software. The parameters were set as follows: the model was p-distance, the missing data method was Partial deletion, the cutoff was 50%, and the Bootstrap was set to 1000 [43]. The online tool iTOL v6 (https://itol.embl.de/, accessed on 12 July 2023) was used to annotate the NJ tree. To further understand the characteristics of TgMADS proteins, this study used Jalview software (version 2.11.2.0)to perform multiple sequence alignment analyses of TgMADS proteins to determine the types of different TgMADS proteins [44].

### 2.4. Distribution Analysis of TgMADS

In this study, the *TgMADS* gene of tulips was determined based on the unigene file constructed from the full-length transcriptome data. According to the location of *TgMADS* family genes in tulips, the distribution of *TgMADS* was mapped by MG2C (http://mg2c.iask.in/mg2c_v2.1/, accessed on 10 July 2023) [45].

### 2.5. Sequence Similarity Analysis of TgMADS Genes

At present, there is no reference genome for tulips, and gene collinearity analysis cannot be carried out. Therefore, blast comparison is carried out according to the sequence similarity between genes to obtain the correlation between genes, which is used to explore the evolutionary relationship between *TgMADS*. Comparison parameters: evalue: 10^−10^, -qcov_hsp_per: 90, -max_hsps 1.

### 2.6. Ka/Ks Analysis of TgMADS Genes

Ka/Ks analysis is one of the most common analyses in bioinformatics, which has important applications in studying the evolution of nucleic acid molecules. In genetics, Ka/Ks represents the ratio between the nonsynonymous substitution rate (Ka) and the synonymous substitution rate (Ks) of two protein-coding genes. This ratio can determine whether there is selective pressure acting on this protein-coding gene. In this study, KaKs_Calculator (version 2.0) software was used to analyze *TgMADS* family genes [46].

### 2.7. Gene Structure and Conserved Motif Analysis TgMADS Genes

By analyzing the distribution of introns/exons and understanding the structural characteristics of the protein family genes, it can be used as a new source of evolutionary information. The software GSDS v2.0 (http://gsds.gao-lab.org/, accessed on 12 July 2023) is used to visualize the gene structure of the gene family members [47].

To better understand the similarity and diversity of conserved motifs of TgMADS family proteins, this study used MEME software (version: v5.0.5) to analyze the conserved motifs of the tulip TgMADS family [48]. The number of parameters for finding conserved motifs was set to 15, and the remaining parameters refer to Mo’s study [49].

### 2.8. Expression Analysis of TgMADS Genes Based on RNA-Seq

To explore the cold induction of *MADS* genes in bulbs, we downloaded RNA-seq data from the public database to reveal the expression of *MADS* genes. After mapping and expression quantitative analysis, the expression information of 32 *TgMADS* members was obtained, and the expression heatmap was drawn by R. The RNA-seq data can be obtained from http://www.ncbi.nlm.nih.gov/bioproject/327809, accessed on 12 July 2023 [50].

### 2.9. Expression Analysis of TgMADS Genes Based on qRT-PCR

An RNA extraction kit (BioRun Biosciences Co., Ltd., Wuhan, China) was used to extract total RNA from the materials preserved in 2.1 (each extraction used 0.1 g samples), and cDNA was synthesized (100 ng RNA for each synthesis) using a reverse transcription kit (Youcan Biosciences Co., Ltd., Shanghai, China). Nine *TgMADS* genes that showed good cold response ability in the transcriptome were selected for qRT-PCR analysis, and the *Tgactin* gene was used as the control for normalization. Each reaction contained 7 µL SYBR qPCR Master Mix (Youcan Biosciences Co., Ltd., Shanghai, China), 1 µL cDNA, 0.5 µL F-primer, 0.5 µL R-primer, and ddH_2_O to 20 µL. The primers used in this study are shown in Table S1, and primer 5 software was used to design the primers [51]. The relative expression level was calculated by the 2^−∆∆Ct^ method.

## 3. Results

### 3.1. Identification of TgMADS Gene Family Members

According to homologous sequence alignment and conserved domain analysis, a total of 32 *TgMADS* members were identified in this study, named *TgMADS1-TgMADS32* (Table 1). The length of 32 TgMADS proteins ranged from 100 aa (TgMADS32) to 336 aa (TgMADS1). The isoelectric point and molecular weight of TgMADS protein were 6.06 (TgMADS29) −11.16 (TgMADS32) and 11462.43Da (TgMADS32) to 37396.38Da (TgMADS1), respectively. According to the distribution of the grand average (GRAVY) from −0.484 (TgMADS27) to −1.029 (TgMADS9), we know that all TgMADSs are hydrophilic proteins. 

Subcellular localization analysis showed that 31 of the 32 TgMADS proteins were located in the nucleus, while only one protein (TgMADS1) was located in the extracellular space.

In order to explore whether *TgMADS* gene family proteins have transmembrane transport function, this study also used DeepTMHMM (version: 1.0.8), a software based on a deep learning model, to predict transmembrane helices. It combines the hydrophobicity of the transmembrane region, charge bias, helix length, and topological limitation of membrane proteins and predicts the transmembrane region and inside and outside the membrane as a whole to determine whether a protein is a membrane protein. The results showed that the predicted probability of amino acids of all 32 TgMADS proteins was 100%, indicating that the proteins encoded by the *TgMADS* gene family do not have the biological function of participating in transmembrane transport.

In addition, this study also used SignalP (version v5.0b) software to predict the signal peptide of the *TgMADS* gene family. The software can predict whether there are potential signal peptide cleavage sites and their locations in a given amino acid sequence. According to the results, the presence of signal peptides was not predicted in the entire *TgMADS* gene family.

### 3.2. Phylogenetic Analysis of the TgMADS Gene Family

**Figure 1:**
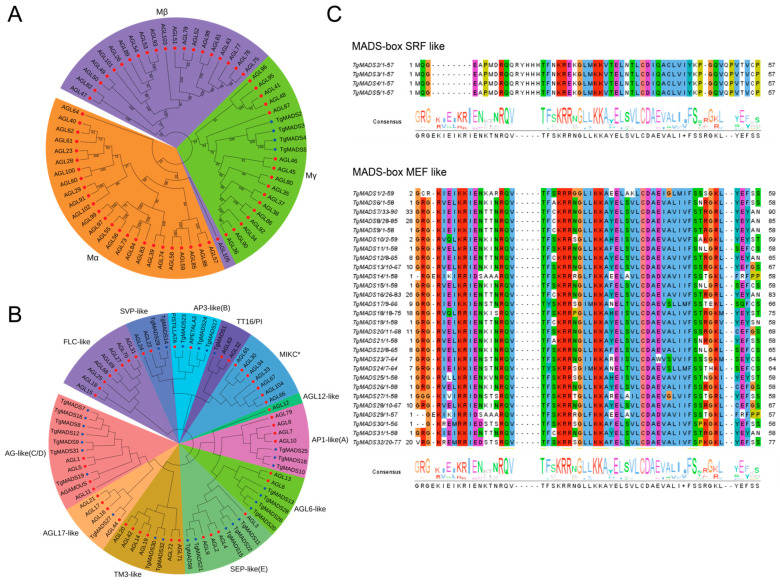
The protein sequence analysis of TgMADS proteins. (**A**) Phylogenetic analysis results of TgMADS proteins, type I. The phylogenetic tree was constructed based on the identified TgMADS proteins and the MADS proteins of *A. thaliana*. Figure 1A is a protein belonging to MADS gene type I, in which three colors correspond to Mα, Mβ, and Mγ, respectively. (**B**) Phylogenetic analysis results of TgMADS proteins, type II. The phylogenetic tree was constructed based on the identified TgMADS protein and the *A. thaliana* MADS protein. Figure 1B is a protein belonging to the *MADS* gene type II, of which 12 colors correspond to AG-like (C/D), FLC-like, SVP-like, TT16/PI, MIKC*, SEP-like (E), TM3-like, AP1-like (A), AP3-like (B), AGL6-like, AGL12-like, and AGL17-like, a total of 12 subfamilies. (**C**) Multiple sequence alignment results of the *TgMADS* gene family. The identified 32 TgMADS proteins were divided into two categories: type I (SRF-like) and type II (MEF-like). Different colors represent the consistency of amino acids.

In order to more systematically understand the evolution and phylogenetic relationship of tulip MADS proteins, we introduced the Arabidopsis MADS protein sequence and the identified TgMADS protein sequence to construct a phylogenetic tree. The results showed that there were two groups of MADS proteins in all members involved in the construction, namely MADS protein type I (Figure 1A) and MADS protein type II (Figure 1B). In the MADS type I protein, TgMADS2-TgMADS5 and some Arabidopsis MADS proteins are divided into three subfamilies: Mα, Mβ, and Mγ. The Mα subfamily contains 25 Arabidopsis MADS proteins and does not contain TgMADS proteins; Mβ contains 21 Arabidopsis MADS proteins and does not contain TgMADS proteins; Mγ contains 15 Arabidopsis MADS proteins and 5 tulip TgMADS proteins. Four tulip TgMADS proteins (TgMADS2-TgMADS5) in MADS protein type I are present in the Mγ subfamily.

All the remaining TgMADS proteins and all the remaining Arabidopsis MADS proteins were clustered into MADS protein type II by a clustering relationship. Among them, AG-like (C/D) subfamily proteins have the function of regulating floral organ differentiation and determining flowering [9]. FLC-like subfamily proteins have the function of inhibiting flowering [52]. This subfamily contains eight Arabidopsis MADS proteins and does not contain TgMADS proteins. The SVP-like subfamily is also an important flowering suppressor gene [53], which contains two Arabidopsis MADS proteins and two TgMADS proteins; tT16/PI subfamily proteins are involved in the accumulation of proanthocyanidins in the endodermis [54]. The subfamily contains two Arabidopsis MADS proteins and one TgMADS protein. MIKC* subfamily proteins are involved in the regulation of pollen maturation and germination [55]. The subfamily contains seven Arabidopsis MADS proteins and does not contain TgMADS proteins; SEP-like (E) subfamily proteins play an important role in pollen maturation and pollen tube formation [56]. This subfamily contains four Arabidopsis MADS proteins and five TgMADS proteins. TM3-like subfamily proteins are regulators of flower branching [9]. This subfamily contains six Arabidopsis MADS proteins and two TgMADS proteins. AP1-like (A) subfamily proteins not only play a central role in the regulatory network of plant floral induction but also determine the formation of floral organs [57]. This subfamily contains four Arabidopsis MADS proteins and three TgMADS proteins. AP3-like (B) subfamily proteins are involved in the development of stamens [58]. This subfamily contains two Arabidopsis MADS proteins and three TgMADS proteins. AGL6-like subfamily proteins are involved in regulating the development of floral organs and floral meristems [9]. This subfamily contains two Arabidopsis MADS proteins and four TgMADS proteins. AGL12-like subfamily proteins play an important role in root development and flowering transition [9]. This subfamily contains one Arabidopsis MADS protein and does not contain the TgMADS protein. AGL17-like subfamily proteins play an important role in plant photoperiod [9]. This subfamily contains four Arabidopsis MADS proteins and one TgMADS protein.

In order to further determine the similarity between the TgMADS domains of tulips, 32 TgMADS domain sequences were compared in this study (Figure 1C). The results showed that there were 4 type I (SRF-like) *TgMADS* genes and 28 type II (MEF-like) TgMADS proteins, and they were highly conserved, with type I (SRF-like) being more conserved in the tulips.

### 3.3. Localization and Collinearity Analysis of TgMADS Gene

**Figure 2:**
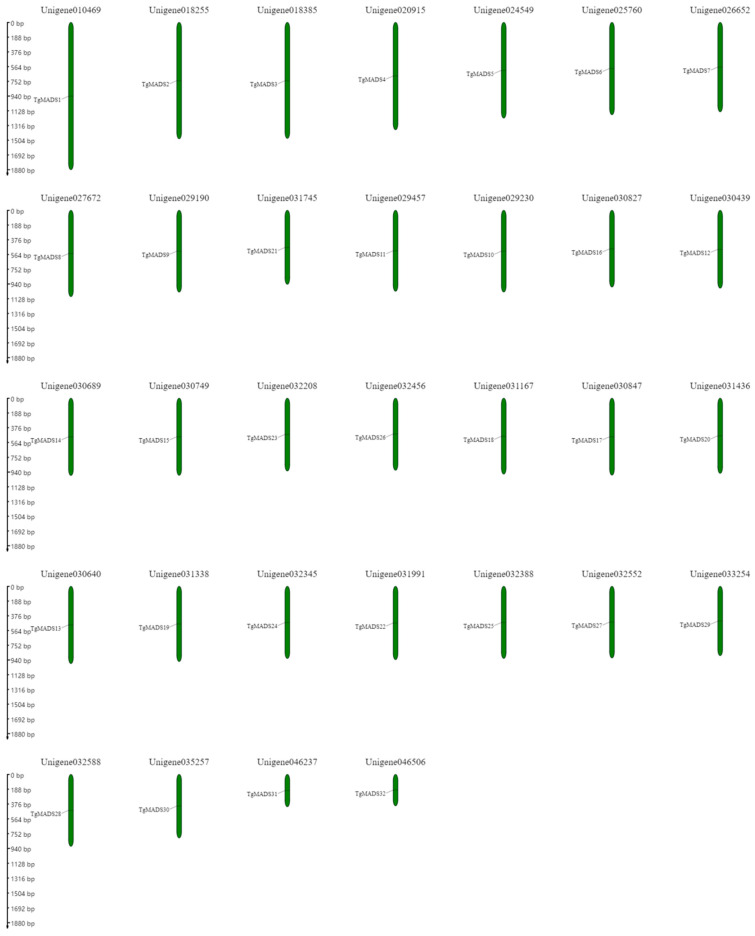
The distribution of *TgMADS* genes on chromosomes. The scale bar on the left is used to measure the length of the unigene.

In this study, 32 unigenes were found to be *MADS* genes using full-length transcriptome assembly technology. They were unigene010469, unigene018255, unigene018385, unigene020915, unigene024549, unigene025760, unigene026652, unigene026652, unigene026652, unigene031745, unigene029457, unigene029230, unigene030827, unigene030439, unigene030689, unigene030689, unigene032208, unigene032456, unigene031167, unigene031167, unigene031436, unigene031436, unigene031338, unigene031338, unigene031338, unigene032552, unigene033254, unigene032588, unigene035257, unigene035257, and unigene046506. The specific distribution is shown in Figure 2.

**Figure 3:**
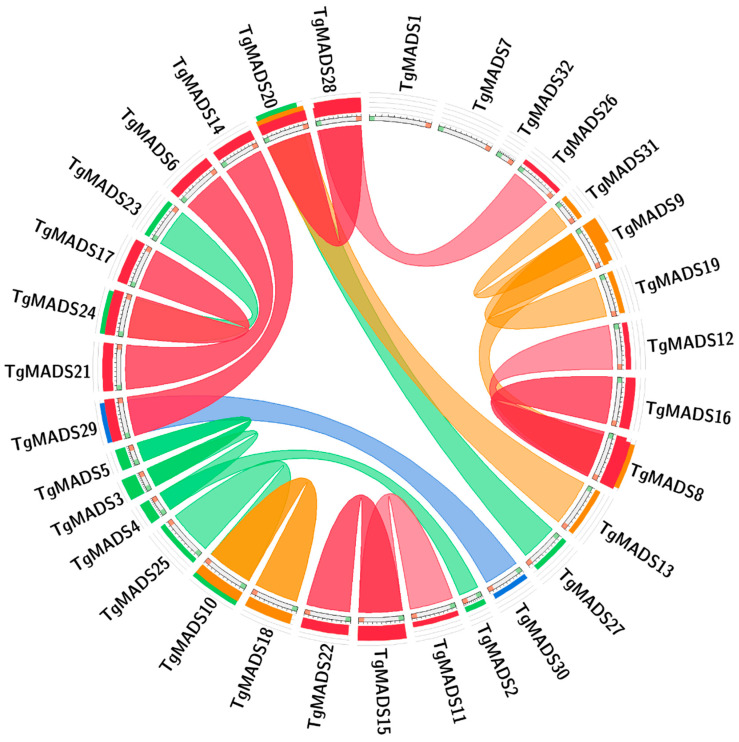
The results of collinearity analysis of *TgMADS* gene in tulip. Genes connected by lines are genes with highly similar relationships.

In order to explore the evolutionary relationship between *TgMADS* genes in tulips, this study obtained the correlation between genes by blast comparison of the sequence similarity between all *TgMADS* genes. The results are shown in Figure 3. Through analysis, it was found that there were 21 pairs of fragment repeats among the 32 *TgMADS* genes, among which *TgMADS1*, *TgMADS7*, and *TgMADS32* genes did not have any repeat relationship with *TgMADS* genes. Since each of the identified 32 *TgMADS* genes is distributed on the corresponding different unigenes, *TgMADS* genes do not have any gene pairs with tandem repeats.

In genetics, Ka/Ks analysis plays an important role in studying the evolution of nucleic acid molecules. The results showed that there were five pairs of genes with Ka/Ks values greater than or equal to 1: *TgMADS12-TgMADS8*, *TgMADS16-TgMADS8*, *TgMADS26-TgMADS28*, *TgMADS12-TgMADS16*, and *TgMADS12-TgMADS7*. Among them, four pairs of genes (*TgMADS12-TgMADS8*, *TgMADS16-TgMADS8*, *TgMADS26-TgMADS28*, and *TgMADS12-TgMADS16*) had Ka/Ks values greater than 1, which were 50, 50, 50, and 47.3832, respectively. Except for the above five pairs of genes, the Ka/Ks values of other gene pairs were less than 1 (Table S2).

### 3.4. Analysis of TgMADS Gene Structure and Conserved Motifs

**Figure 4:**
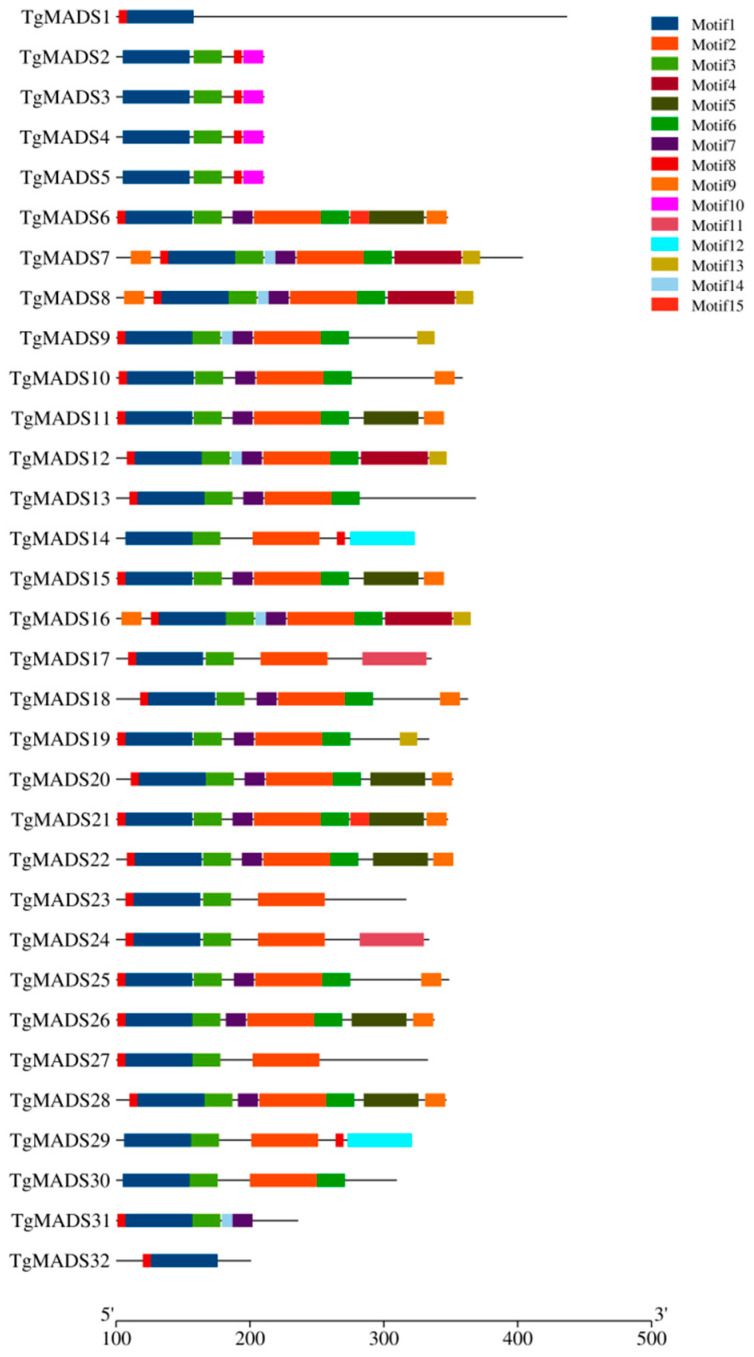
The conserved motifs of TgMADS proteins. Different color boxes represent different conserved motifs.

Motif 1 exists in all TgMADS proteins, while motif 3 is distributed on all TgMADS proteins except TgMADS1 and TgMADS32. Combined with the results of gene collinearity analysis, this may be the reason why TgMADS1 and TgMADS32 do not have any genes related to replication in tulips. In addition to the above-conserved motifs, there are also some specific conserved motifs, some of which exist in a single TgMADS protein, and the other part also shows conservation among some TgMADS proteins. For example, motif 8 and motif 10 are distributed on TgMADS2-TgMADS5, and these four proteins are members of the type I Mγ subfamily of *MADS* genes, while motif 12 only exists on TgMADS14 and TgMADS29. In phylogenetic analysis, TgMADS14 and TgMADS29 belong to the type II SVP-like subfamily of *MADS* genes. These phenomena all point to the fact that TgMADS protein members of the same subfamily may have similar biological functions due to their similar motif distribution (Figure 4).

**Figure 5:**
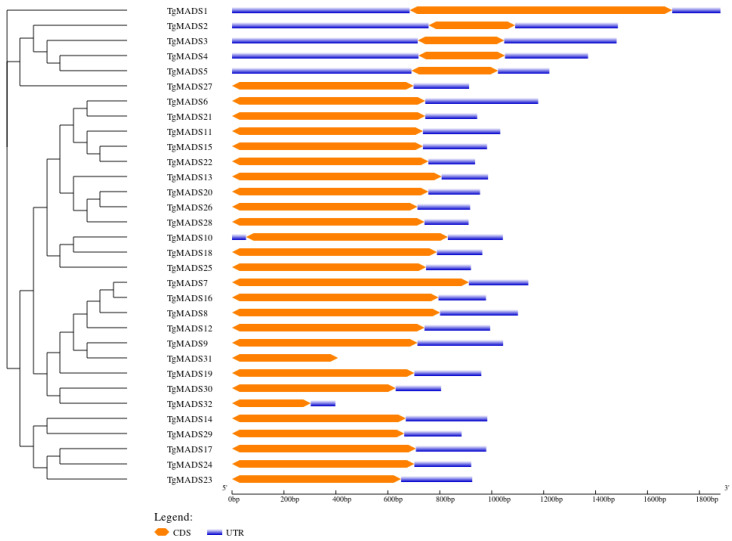
Gene structural analysis of *TgMADS* genes. The yellow box is the coding region (CDS), and the blue box is the non-coding region (UTR).

Gene structure is an important factor determining the relationship between genome evolution and functional differentiation of multigene family members. The analysis results (Figure 5) showed that among the 32 *TgMADS* genes, only the *TgMADS31* gene did not contain non-coding regions, and approximately 18.8% (6/32) of *TgMADS* genes: *TgMADS1*, *TgMADS2*, *TgMADS3*, *TgMADS4*, *TgMADS5*, and *TgMADS10* contained 5′ and 3′ non-coding regions, and the remaining 78.1% (25/32) of *TgMADS* genes only contained 3′ non-coding regions.

### 3.5. TgMADS Gene Expression Pattern Analysis

**Figure 6:**
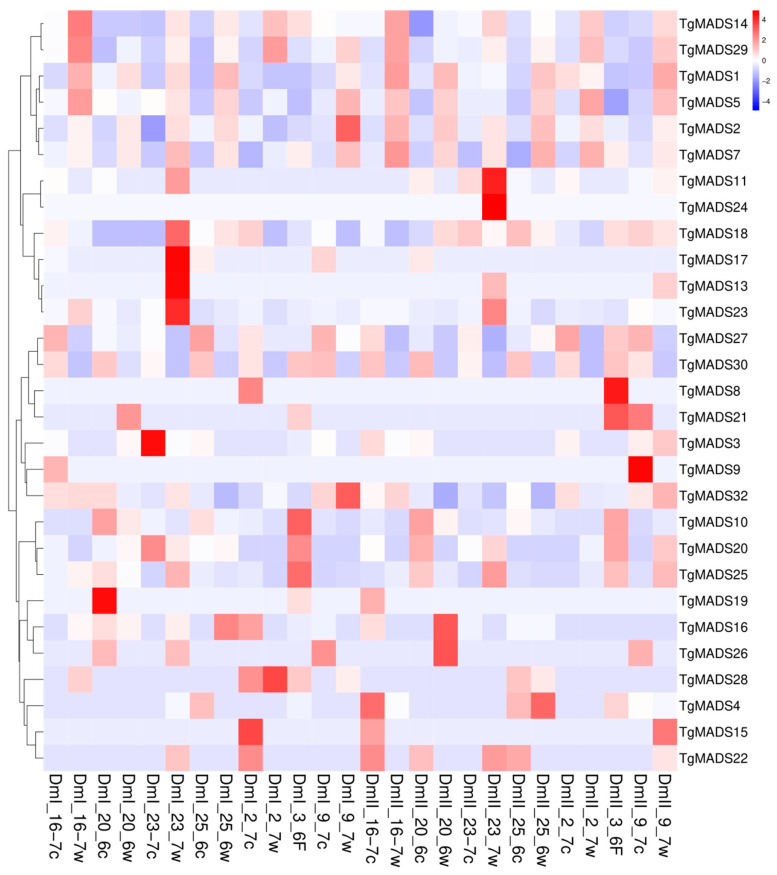
Transcriptome expression pattern analysis of 32 *TgMADS* genes in tulip bulb meristems under normal temperature (19 °C) and low-temperature (8–9 °C) treatments. The abscissa represents the name of the sample under normal temperature (19 °C) and low temperature (8–9 °C) treatments, where DmI is the first group of biological repetitions, DmII is the second group of biological repetitions, the sample w tail is normal temperature treatment, and the c tail is low-temperature treatment; the ordinate represents the identified *TgMADS* family genes and the clustering results of the genes; color represents the expression level of the gene in the sample, and red to blue represents the expression level of the gene from high to low.

In the expression pattern analysis (Figure 6), the samples rich in meristem from seven individual tulip bulbs were mixed together to form a biological repeat, and the selected *TgMADS* family was treated at room temperature (19 °C and low temperature (8–9 °C). The expression genes were subjected to hierarchical clustering analysis, and the genes with the same or similar expression behavior were clustered. In this experiment, two sets of biological repeats, DmI and DmII, were performed, in which the sample w tail was labeled as the room temperature treatment and the c tail was labeled as the low-temperature treatment. As shown in Figure 6, the downregulated genes at low-temperature treatment (8–9 °C) are *TgMADS14*, *TgMADS29*, *TgMADS1*, *TgMADS5*, *TgMADS2*, *TgMADS7*, *TgMADS10*, *TgMADS20*, and *TgMADS25*.

*TgMADS27* and *TgMADS30* showed an overall upregulation trend during most of the low-temperature treatment. In addition to the above genes, the remaining *TgMADS* family members did not show regular expression changes with temperature changes. Based on this result, it is impossible to infer the changes in other gene functions affected by temperature.

### 3.6. qRT-PCR Analysis of TgMADS Genes under Cold Treatment

**Figure 7:**
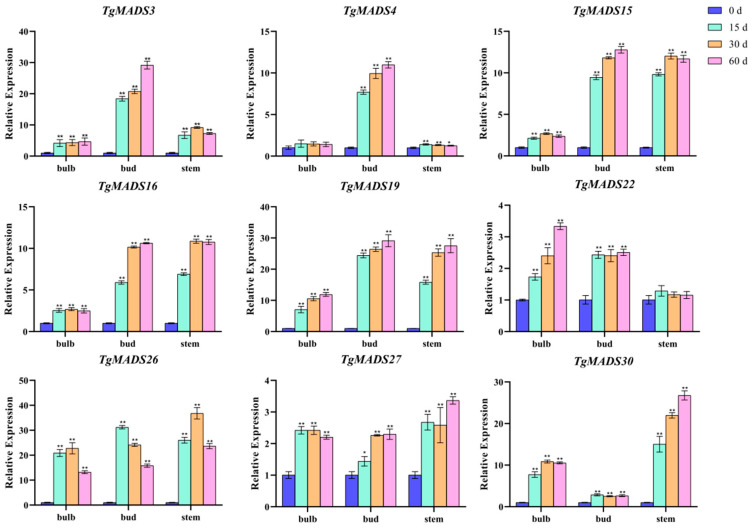
qRT-PCR analysis of nine *TgMADS* genes under cold treatment. ANOVA analysis was used to test significance (*p* < 0.05 indicated by *, *p* < 0.001 indicated by **).

The transcriptome data reveal the expression pattern of *TgMADS* genes in response to low temperatures in bulbs. In this study, nine *TgMADS* genes with good responses to low temperature were selected, which were *TgMADS3*, *TgMADS4*, *TgMADS15*, *TgMADS16*, *TgMADS19*, *TgMADS22*, *TgMADS26*, *TgMADS27*, and *TgMADS30*. These genes were tested by qRT-PCR in the bulb, bud, and stem tissues to explore their expression patterns. As shown in Figure 7, the expression levels of *TgMADS3* and *TgMADS4* in buds were significantly higher than those in bulbs and stems, and the expression of both in buds always showed an upward trend with the prolongation of cold treatment time. *TgMADS15* and *TgMADS16* also showed similar expression patterns, both of which had strong cold response ability in buds and stems (upregulated by more than 10 times). *TgMADS19* has good cold response ability in bulbs, buds, and stems. The upregulation of *TgMADS22* and *TgMADS27* in bulbs, buds, and stems was less than four times in 0 to 60 days, indicating that the response of the two genes to cold was weak. The response of *TgMADS26* to cold treatment showed a trend of increasing first and then decreasing, no matter in which tissue. The expression of *TgMADS30* in flowers was not significantly upregulated. All *TgMADS* genes showed different response patterns to cold treatment, which also proved that the response of *TgMADS* genes to cold treatment was tissue-specific.

## 4. Discussion

The *MADS* gene family encodes transcription factors that are widely involved in regulating plant growth and development, including flower, seed, and root development, as well as morphological diversity. Therefore, understanding the structure and function of *MADS* genes is crucial for understanding how plant growth and development are regulated. Up to now, the *MADS* gene family has been studied in many plants, including watermelon [11], apple [12], cabbage [13], wheat [14], maize [15], cotton [8], chrysanthemum [9], orchid [17], etc. However, the related research on the *MADS* gene family found in tulips is not sufficient. In this study, the *MADS* gene of tulips was identified in the whole genome, and its gene structure and genetic evolution were discussed. The expression difference in the *MADS* gene in the tulip bulb meristem after room temperature (19 °C) and low-temperature (8–9 °C) treatment was discussed. This study provides an effective argument for exploring the above-mentioned molecular processes and promoting tulip resistance breeding.

In this study, a total of 32 *MADS* gene family members were identified in tulips. According to subcellular localization analysis, it was found that all TgMADS proteins were localized to the nucleus except TgMADS1, which indicated that TgMADS was specifically accumulated in the nucleus. This phenomenon is not only similar to other plant MADS-box proteins but also consistent with the role of MADS-box proteins as transcription factors. Some transcription factors containing MADS-box have been shown to be localized in the nucleus [59,60,61,62]. It is worth noting that [63] studied the subcellular localization of NnMADS proteins in lotus flowers. It was found that except for NnMADS1 located on the cell membrane, other NnMADS proteins were located in the nucleus, which was very similar to the subcellular localization results of TgMADS proteins found in this study. 

In addition, this study also predicted the transmembrane transport function and signal peptide of the *TgMADS* gene family. The results showed that no protein members with transmembrane transport function and possible signal peptide molecules were found. Due to the existence of no reference genome, this study identified the unigene and location of these 32 *MADS* genes by full-length transcriptome splicing.

Through the clustering of MADS proteins in tulips and *A. thaliana* MADS proteins, they can be divided into two types (*MADS* gene type I and *MADS* gene type II) and 15 subfamilies. Among them, *MADS* gene type I contains three subfamilies (Mα, Mβ, and Mγ). *MADS* gene type II includes 12 subfamilies (AG-like (C/D), FLC-like, SVP-like, TT16/PI, MIKC*, SEP-like €, TM3-like, AP1-like (A), AP3-like (B), AGL6-like, AGL12-like, and AGL17-like). According to previous studies, these 12 subfamilies play an important regulatory role in the growth and development of multiple plants. For example, AG-like (C/D) subfamily proteins have the function of regulating floral organ differentiation and determining flowering [7], FLC-like subfamily and SVP-like subfamily proteins have the function of inhibiting flowering [64,65], TT16/PI subfamily proteins are involved in the accumulation of proanthocyanidins in the endodermis [66], MIKC* subfamily and SEP-like (E) subfamily proteins are involved in the regulation of pollen maturation and germination and pollen tube formation, TM3-like subfamily proteins are regulators of flower branching [7], AP1-like (A) subfamily proteins not only play a central role in the regulatory network of plant flowering induction, but also determine the formation of floral organs [57], and AP3-like (B) subfamily proteins are involved in stamen development [7]. The *MADS* gene type I Mα, Mβ subfamily, and *MADS* gene type II AGL12-like, MIKC*, and FLC-like subfamily gene members are missing in tulips. Gene structure analysis showed that there were significant differences in the structure and sequence length of *TgMADS* genes in different subfamilies. Only 18.8% (6/32) of the genes had 5′ non-coding regions, and the remaining gene members only had 3′ non-coding regions. The observed discrepancies may arise from the transcriptomic, rather than the genomic, basis of the present study, which could result in an incomplete representation of UTR in the assembled unigene sequences. In addition, the study found that *TgMADS2*, *TgMADS3*, *TgMADS4*, and *TgMADS5* genes have very similar structures. These four genes are in the same subfamily and are highly collinear in both phylogenetic analysis and collinearity analysis. This indicates that the members with high homology have similar non-coding region and coding region distribution structures, and the gene structure of Mγ subfamily members is more conservative than that of other subfamily members.

Through the analysis of conserved motifs, it was found that all TgMADS proteins have motif 1; motif 2 is present in all *MADS* gene type II (except *TgMADS31* and *TgMADS32*) member proteins. Based on this result, motif 2 is a highly conserved K-box domain, which was previously confirmed in the conserved motif analysis of watermelon and melon MADS gene family. In addition to TgMADS1 and TgMADS32, there is a motif 3 distributed on all the remaining TgMADS proteins. Combined with the results of gene collinearity analysis, this may be the reason why *TgMADS1* and *TgMADS32* do not have any replication genes in tulips. The domain of motif 8 and motif 10 was found only in the four member proteins of *MADS* gene type I, which was very conservative in this group. The results indicated that motif 8 and motif 10 were special conserved motifs belonging to the Mγ subfamily. The motif structure of each subfamily is relatively similar, indicating that the members of each subfamily may have a similar function.

Studies have shown that the evolutionary process of genes and paragenes has an important relationship with the function of gene families [64]. In this study, 32 *TgMADS* genes in the whole genome of tulips were determined. The identification results based on fragment repeats and tandem repeats showed that there were 21 pairs of fragment repeats among the 32 *TgMADS* genes found, among which *TgMADS1*, *TgMADS7*, and *TgMADS32* genes did not have any *TgMADS* genes with repetitive relationships. Combined with the phylogenetic clustering results of TgMADS proteins previously investigated, except for the *TgMADS20* and *TgMADS27* fragment repeat gene pairs that do not belong to the same subfamily of phylogenetic analysis results (*TgMADS20* belongs to the *MADS* gene type II AGL6-like subfamily, *TgMADS27* belongs to the MADS gene type II AGL17-like subfamily), all the remaining fragment repeat gene pairs are in the same phylogenetic analysis results subfamily. The results of these two analyses provide strong and clear evidence for exploring the evolutionary relationship of different *TgMADS* genes. Since all *TgMADS* genes exist on different genes, there is no tandem repeat relationship in tulip *MADS* genes.

In the Ka/Ks analysis, five pairs of genes (*TgMADS12-TgMADS8*, *TgMADS16-TgMADS8*, *TgMADS26-TgMADS28*, *TgMADS12-TgMADS16*, and *TgMADS12-TgMADS7*) had Ka/Ks values greater than 1. Among them, four pairs of genes (*TgMADS12-TgMADS8*, *TgMADS16-TgMADS8*, *TgMADS26-TgMADS28*, and *TgMADS12-TgMADS16*) had Ka/Ks values far greater than 1, 50, 50, 50, and 47.3832, respectively. The above results indicate that these five pairs of genes have positive selection effects in the evolutionary process, and the first four pairs of genes have strong positive selection effects. These four pairs of genes are genes that have rapidly evolved recently [65], which are of great significance for the evolution of species. Except for the above five pairs of genes, other gene pairs were affected by purification selection during evolution.

In this study, the samples rich in meristem from seven individual tulip bulbs were mixed together to form a biological replicate. After preparing two sets of biological replicate materials, they were treated at room temperature (19 °C) and low temperatures (8–9 °C), respectively, and the selected *TgMADS* family expression genes were subjected to hierarchical clustering analysis. Among the downregulated genes (*TgMADS14*, *TgMADS29*, *TgMADS1*, *TgMADS5*, *TgMADS2*, *TgMADS7*, *TgMADS10*, *TgMADS20*, and *TgMADS25*) at low-temperature treatment (8–9 °C), *TgMADS5* and *TgMADS2* belong to the type I Mγ subfamily of *MADS* genes. *TgMADS7* is the only *TgMADS* member belonging to the *MADS* gene type II TT16/PI. *TgMADS14* and *TgMADS29* belong to the type II SVP-like subfamily of *MADS* genes, which are important flowering inhibitory genes. Based on this result, the low-temperature treatment may weaken the expression of flowering inhibitory genes, thereby promoting the flowering of tulips. *TgMADS10* and *TgMADS25* belong to the AP1-like (A) subfamily of *MADS* gene type II. This result indicated that low temperatures will affect the flowering induction of tulips and inhibit the formation of floral organs. Among the upregulated genes (*TgMADS27* and *TgMADS30*) under low-temperature treatment (8–9 °C), *TgMADS30* belongs to the *MADS* gene type II TM3-like subfamily. Based on this result, the relationship between flower branching and temperature changes can be further studied. It is worth noting that *TgMADS27* has a collinear relationship with *TgMADS20* downregulated at low-temperature treatment, but the expression of the two affected by temperature is completely opposite, and the reason needs to be further explored. *TgMADS27* is the only *TgMADS* member belonging to the type II AGL17-like subfamily of the *MADS* gene. The low temperature may affect the photoperiod of tulips.

A previous study identified 167 *MADS* gene members in rapeseed and analyzed their expression under low-temperature stress [66]. They belong to the TM3-like subfamily (five members), FLC-like (three members), AGL17-like subfamily (one member), and Mα subfamily (two members), respectively. Combined with the results of this study and this result, it was found that the genes responding to low-temperature stress mainly belong to *MADS* gene type II, and a small amount belongs to *MADS* gene type I. In addition, the TM3-like subfamily and AGL17-like subfamily are subfamilies that respond to cold stress in both crops. The above two subfamilies are highly conserved during long-term evolution.

*TgMADS3* and *TgMADS4* genes showed a continuous upward trend in bud tissues under low-temperature treatment, indicating that they may be involved in the development of flower tissues under low-temperature conditions. These two genes are worthy of further functional verification to explore their specific roles in the process of flowering. *TgMADS15* and *TgMADS16* were highly sensitive to low-temperature treatment in bud and stem tissues, and their expression levels were significantly upregulated by more than 10 times. It indicates that these two genes may be important low-temperature response factors in tulips and participate in the adaptation of tulips to low-temperature environments. The next step is to study the effects of overexpression or deletion of these two genes on the low-temperature adaptability and flower development of tulips. The *TgMADS19* gene showed a good response to low-temperature treatment in the three tissues of tulips, indicating that it may be a systemic low-temperature response factor and participate in regulating the systematic adaptation of tulips to low temperatures. It is worth noting whether *TgMADS19* also affects the flowering period of tulips. The response of *TgMADS22* and *TgMADS27* to low temperature was weak, indicating that they may not be the key genes in the process of low-temperature adaptation of tulips. However, some of its specific roles in low-temperature adaptation cannot be completely ruled out. The low-temperature response patterns of *TgMADS26* and *TgMADS30* are special, and further research is needed to explore their complex expression regulation mechanisms.

In summary, this study revealed that certain *MADS-box* genes in tulips, including *TgMADS15/16* and *TgMADS19*, are differentially expressed under low-temperature conditions and may play important roles in the vernalization response pathway. These results advance our understanding of the molecular mechanisms regulating cold-induced flowering in geophytes. Further functional characterization of the identified MADS-box candidates could elucidate their precise roles in transducing vernalization cues and inform molecular breeding efforts to improve traits such as chilling tolerance and flowering time in tulips. This work lays a foundation for continued investigation into the genes and pathways governing vernalization, a pivotal yet poorly characterized process in geophyte flowering and productivity.

## Figures and Tables

**Table 1:** Physicochemical properties of *TgMADS* gene family members.

Gene Name	Subcellular Localization	Protein Length (aa)	Molecular Weight (Da)	Isoelectric Point	Stability Index	Liposolubility Index	GRAVY
*TgMADS1*	Intercellular space	336	37,396.38	7.54	53.86	81.34	−0.625
*TgMADS2*	nucleus	110	12,828.82	9.35	50.10	77.91	−0.642
*TgMADS3*	nucleus	110	12,828.82	9.35	50.10	77.91	−0.642
*TgMADS4*	nucleus	110	12,828.82	9.35	50.10	77.91	−0.642
*TgMADS5*	nucleus	110	12,828.82	9.35	50.10	77.91	−0.642
*TgMADS6*	nucleus	247	27,919.65	9.16	52.28	78.70	−0.655
*TgMADS7*	nucleus	303	35,098.54	8.94	59.48	70.86	−0.889
*TgMADS8*	nucleus	266	30,840.78	9.15	56.99	73.38	−0.878
*TgMADS9*	nucleus	237	27,825.35	9.33	61.48	68.73	−1.029
*TgMADS10*	nucleus	258	29,553.34	8.95	57.47	73.80	−0.909
*TgMADS11*	nucleus	244	27,871.45	7.02	52.36	79.14	−0.72
*TgMADS12*	nucleus	246	28,678.39	9.06	55.32	75.37	−0.855
*TgMADS13*	nucleus	268	30,635.63	9.07	48.64	73.88	−0.799
*TgMADS14*	nucleus	222	25,059.44	6.43	59.46	88.74	−0.609
*TgMADS15*	nucleus	244	27,881.48	7.07	49.34	79.14	−0.721
*TgMADS16*	nucleus	264	30,754.68	9.16	56.43	73.94	−0.884
*TgMADS17*	nucleus	235	26,910.46	9.18	35.68	75.49	−0.784
*TgMADS18*	nucleus	262	30,382.42	8.48	69.94	79.27	−0.767
*TgMADS19*	nucleus	233	26,757.12	9.28	45.40	79.61	−0.713
*TgMADS20*	nucleus	251	28,962.96	9.14	47.27	75.78	−0.757
*TgMADS21*	nucleus	247	27,919.65	9.16	52.28	78.70	−0.655
*TgMADS22*	nucleus	251	28,627.34	7.74	50.81	76.93	−0.747
*TgMADS23*	nucleus	216	25,461.11	9.05	65.98	73.10	−0.965
*TgMADS24*	nucleus	233	26,880.42	8.69	42.52	76.14	−0.791
*TgMADS25*	nucleus	248	28,396.23	6.38	60.33	80.20	−0.698
*TgMADS26*	nucleus	237	27,159.91	8.93	47.53	80.25	−0.662
*TgMADS27*	nucleus	232	26,620.5	8.58	51.10	102.03	−0.484
*TgMADS28*	nucleus	246	28,341.24	9.08	46.61	77.32	−0.744
*TgMADS29*	nucleus	220	24,846.2	6.06	59.73	89.55	−0.592
*TgMADS30*	nucleus	209	24,012.38	6.20	75.65	78.42	−0.765
*TgMADS31*	nucleus	136	15,554.62	9.80	45.92	77.50	−0.857
*TgMADS32*	nucleus	100	11,462.43	11.16	65.23	74.10	−0.673

## Data Availability

Data are contained within the article or Supplementary Materials.

## Supplementary Materials

The following supporting information can be downloaded at: https://www.mdpi.com/article/10.3390/genes14101974/s1, Table S1: *TgMADS* qRT-PCR primers; Table S2: KaKs Stat of *TgMADS*.

## References

[1] Rietveld P.L., Wilkinson C., Franssen H.M., Balk P.A., van der Plas L.H., Weisbeek P.J., Douwe de Boer A. (2000). Low temperature sensing in tulip (*Tulipa gesneriana* L.) is mediated through an increased response to auxin. J. Exp. Bot..

[2] Mondragón-Palomino M. (2013). Perspectives on MADS-box expression during orchid flower evolution and development. Front. Plant Sci..

[3] Saedler H., Becker A., Winter K.-U., Kirchner C., Theißen G. (2001). MADS-box genes are involved in floral development and evolution. Acta Biochim. Pol..

[4] Ng M., Yanofsky M.F. (2001). Function and evolution of the plant MADS-box gene family. Nat. Rev. Genet..

[5] Riechmann J.L., Meyerowitz E.M. (1997). MADS domain proteins in plant development. Biol. Chem..

[6] Münster T., Wingen L.U., Faigl W., Werth S., Saedler H., Theißen G. (2001). Characterization of three GLOBOSA-like MADS-box genes from maize: Evidence for ancient paralogy in one class of floral homeotic B-function genes of grasses. Gene.

[7] Gramzow L., Theißen G. (2013). Phylogenomics of MADS-box genes in plants—Two opposing life styles in one gene family. Biology.

[8] Ren Z., Yu D., Yang Z., Li C., Qanmber G., Li Y., Li J., Liu Z., Lu L., Wang L. (2017). Genome-wide identification of the MIKC-type MADS-box gene family in *Gossypium hirsutum* L. unravels their roles in flowering. Front. Plant Sci..

[9] Becker A., Theißen G. (2003). The major clades of MADS-box genes and their role in the development and evolution of flowering plants. Mol. Phylogenet. Evol..

[10] Kumar K., Srivastava H., Das A., Tribhuvan K.U., Durgesh K., Joshi R., Sevanthi A.M., Jain P.K., Singh N.K., Gaikwad K. (2021). Identification and characterization of MADS box gene family in pigeonpea for their role during floral transition. 3 Biotech.

[11] Wang P., Wang S., Chen Y., Xu X., Guang X., Zhang Y. (2019). Genome-wide analysis of the MADS-box gene family in Watermelon. Comput. Biol. Chem..

[12] Tian Y., Dong Q., Ji Z., Chi F., Cong P., Zhou Z. (2015). Genome-wide identification and analysis of the MADS-box gene family in apple. Gene.

[13] Sheng X.G., Zhao Z.Q., Wang J.S., Yu H.F., Shen Y.S., Zeng X.Y., Gu H.H. (2019). Genome wide analysis of MADS-box gene family in *Brassica oleracea* reveals conservation and variation in flower development. J. BMC Plant Biol..

[14] Ma J., Yang Y., Luo W., Yang C., Ding P., Liu Y., Qiao L., Chang Z., Geng H., Wang P. (2017). Genome-wide identification and analysis of the MADS-box gene family in bread wheat (*Triticum aestivum* L.). PLoS ONE.

[15] Zhao D., Chen Z., Xu L., Zhang L., Zou Q. (2021). Genome-Wide analysis of the MADS-Box gene family in maize: Gene structure, evolution, and relationships. Genes.

[16] Won S.Y., Jung J.-A., Kim J.S. (2021). Genome-wide analysis of the MADS-Box gene family in Chrysanthemum. Comput. Biol. Chem..

[17] Lu H., Liu Z., Lan S. (2019). Genome sequencing reveals the role of MADS-box gene families in the floral morphology evolution of orchids. Hortic. Plant J..

[18] Kanno A., Hirai M., Ochiai T., Simon H., Theissen G. Reduced expression of DEFICIENS-like genes in the sepaloid tepals of viridiflora tulips. Proceedings of the XXVII International Horticultural Congress-IHC2006: International Symposium on Structural and Functional Genomics of 763.

[19] Hirai M., Ochiai T., Kanno A. (2010). The expression of two DEFICIENS-like genes was reduced in the sepaloid tepals of viridiflora tulips. Breed. Sci..

[20] Kanno A., Nakada M., Akita Y., Hirai M. (2007). Class B gene expression and the modified ABC model in nongrass monocots. Sci. World J..

[21] Li C., Wang Y., Xu L., Nie S., Chen Y., Liang D., Sun X., Karanja B.K., Luo X., Liu L. (2016). Genome-wide characterization of the MADS-box gene family in radish (*Raphanus sativus* L.) and assessment of its roles in flowering and floral organogenesis. Front. Plant Sci..

[22] Theissen G., Melzer R. (2007). Molecular mechanisms underlying origin and diversification of the angiosperm flower. Ann. Bot..

[23] Coen E.S., Meyerowitz E.M. (1991). The war of the whorls: Genetic interactions controlling flower development. Nature.

[24] Silva C.S., Puranik S., Round A., Brennich M., Jourdain A., Parcy F., Hugouvieux V., Zubieta C. (2016). Evolution of the plant reproduction master regulators LFY and the MADS transcription factors: The role of protein structure in the evolutionary development of the flower. Front. Plant Sci..

[25] Zahn L.M., Feng B., Ma H. (2006). Beyond the ABC-model: Regulation of floral homeotic genes. Adv. Bot. Res..

[26] Arora R., Agarwal P., Ray S., Singh A.K., Singh V.P., Tyagi A.K., Kapoor S. (2007). MADS-box gene family in rice: Genome-wide identification, organization and expression profiling during reproductive development and stress. BMC Genom..

[27] van Tunen A.J., Eikelboom W., Angenent G.C. (1993). Floral organogenesis in Tulipa. Flower. Newsl..

[28] Michaels S.D., Amasino R.M. (1999). FLOWERING LOCUS C encodes a novel MADS domain protein that acts as a repressor of flowering. Plant Cell.

[29] Hartmann U., Höhmann S., Nettesheim K., Wisman E., Saedler H., Huijser P. (2000). Molecular cloning of SVP: A negative regulator of the floral transition in Arabidopsis. Plant J..

[30] Liljegren S.J., Ditta G.S., Eshed Y., Savidge B., Bowman J.L., Yanofsky M.F. (2000). SHATTERPROOF MADS-box genes control seed dispersal in Arabidopsis. Nature.

[31] Lawton-Rauh A.L., Alvarez-Buylla E.R., Purugganan M.D. (2000). Molecular evolution of flower development. Trends Ecol. Evol..

[32] Henschel K., Kofuji R., Hasebe M., Saedler H., Münster T., Theißen G. (2002). Two ancient classes of MIKC-type MADS-box genes are present in the moss Physcomitrella patens. Mol. Biol. Evol..

[33] Krogan N., Ashton N. (2000). Ancestry of plant MADS-box genes revealed by bryophyte (*Physcomitrella patens*) homologues. New Phytol..

[34] Winter K.-U., Becker A., Münster T., Kim J.T., Saedler H., Theissen G. (1999). MADS-box genes reveal that gnetophytes are more closely related to conifers than to flowering plants. Proc. Natl. Acad. Sci. USA.

[35] Eddy S.R. (2009). A new generation of homology search tools based on probabilistic inference. Genome Informatics 2009: Genome Informatics Series Vol. 23.

[36] Johnson M., Zaretskaya I., Raytselis Y., Merezhuk Y., McGinnis S., Madden T.L. (2008). NCBI BLAST: A better web interface. Nucleic Acids Res..

[37] Madeira F., Park Y.M., Lee J., Buso N., Gur T., Madhusoodanan N., Basutkar P., Tivey A.R., Potter S.C., Finn R.D. (2019). The EMBL-EBI search and sequence analysis tools APIs in 2019. Nucleic Acids Res..

[38] Mistry J., Bateman A., Finn R.D. (2007). Predicting active site residue annotations in the Pfam database. BMC Bioinform..

[39] Sonnhammer E.L., Eddy S.R., Durbin R. (1997). Pfam: A comprehensive database of protein domain families based on seed alignments. Proteins Struct. Funct. Bioinform..

[40] Hallgren J., Tsirigos K.D., Pedersen M.D., Almagro Armenteros J.J., Marcatili P., Nielsen H., Krogh A., Winther O. (2022). DeepTMHMM predicts α and β transmembrane proteins using deep neural networks. BioRxiv.

[41] Almagro Armenteros J.J., Tsirigos K.D., Sønderby C.K., Petersen T.N., Winther O., Brunak S., von Heijne G., Nielsen H. (2019). SignalP 5.0 improves signal peptide predictions using deep neural networks. Nat. Biotechnol..

[42] Katoh K., Standley D.M. (2013). MAFFT multiple sequence alignment are version 7: Improvements in performance and usability. Mol. Biol. Evol..

[43] Kumar S., Tamura K., Nei M. (1994). MEGA: Molecular evolutionary genetics analysis software for microcomputers. Bioinformatics.

[44] Waterhouse A., Procter J., Martin D.A., Barton G.J. (2005). Jalview: Visualization and analysis of molecular sequences, alignments, and structures. BMC Bioinform..

[45] Chao J., Li Z., Sun Y., Aluko O.O., Wu X., Wang Q., Liu G. (2021). MG2C: A user-friendly online tool for drawing genetic maps. Mol. Hortic..

[46] Wang D., Zhang Y., Zhang Z., Zhu J., Yu J. (2010). KaKs_Calculator 2.0: A toolkit incorporating gamma-series methods and sliding window strategies. Genom. Proteom. Bioinform..

[47] Guo A.-Y., Zhu Q.-H., Chen X., Luo J.-C. (2007). GSDS: A gene structure display server. Yi Chuan = Hereditas.

[48] Bailey T.L., Williams N., Misleh C., Li W.W. (2006). MEME: Discovering and analyzing DNA and protein sequence motifs. Nucleic Acids Res..

[49] Mo F., Xue X., Meng L., Zhang Y., Cui Y., Liu J., Cheng M., Wang P., Lv R., Meng F. (2023). Genome-wide identification and expression analysis of SLAC1 gene family in tomato (*Solanum lycopersicum*) and the function of SlSLAC1–6 under cold stress. Sci. Hortic..

[50] Leeggangers H.A., Nijveen H., Bigas J.N., Hilhorst H.W., Immink R.G. (2017). Molecular regulation of temperature-dependent floral induction in *Tulipa gesneriana*. Plant Physiol..

[51] Lalitha S. (2000). Primer premier 5. Biotech Softw. Internet Rep. Comput. Softw. J. Sci..

[52] Crevillen P., Dean C. (2011). Regulation of the floral repressor gene FLC: The complexity of transcription in a chromatin context. Curr. Opin. Plant Biol..

[53] Liu X., Sun Z., Dong W., Wang Z., Zhang L. (2018). Expansion and functional divergence of the SHORT VEGETATIVE PHASE (SVP) genes in eudicots. Genome Biol. Evol..

[54] Ehlers K., Bhide A.S., Tekleyohans D.G., Wittkop B., Snowdon R.J., Becker A. (2016). The MADS box genes ABS, SHP1, and SHP2 are essential for the coordination of cell divisions in ovule and seed coat development and for endosperm formation in Arabidopsis thaliana. PLoS ONE.

[55] Liu Y., Cui S., Wu F., Yan S., Lin X., Du X., Chong K., Schilling S., Theißen G., Meng Z. (2013). Functional conservation of MIKC*-Type MADS box genes in Arabidopsis and rice pollen maturation. Plant Cell.

[56] Gao X., Liang W., Yin C., Ji S., Wang H., Su X., Guo C., Kong H., Xue H., Zhang D. (2010). The SEPALLATA-like gene OsMADS34 is required for rice inflorescence and spikelet development. Plant Physiol..

[57] Fornara F., Parenicová L., Falasca G., Pelucchi N., Masiero S., Ciannamea S., Lopez-Dee Z., Altamura M.M., Colombo L., Kater M.M. (2004). Functional characterization of OsMADS18, a member of the AP1/SQUA subfamily of MADS box genes. Plant Physiol..

[58] Su K., Zhao S., Shan H., Kong H., Lu W., Theissen G., Chen Z., Meng Z. (2008). The MIK region rather than the C-terminal domain of AP3-like class B floral homeotic proteins determines functional specificity in the development and evolution of petals. New Phytol..

[59] Kaufmann K., Melzer R., Theißen G. (2005). MIKC-type MADS-domain proteins: Structural modularity, protein interactions and network evolution in land plants. Gene.

[60] Shih M.-C., Chou M.-L., Yue J.-J., Hsu C.-T., Chang W.-J., Ko S.-S., Liao D.-C., Huang Y.-T., Chen J.J., Yuan J.-L. (2014). BeMADS1 is a key to delivery MADSs into nucleus in reproductive tissues-De novo characterization of *Bambusa edulis* transcriptome and study of MADS genes in bamboo floral development. BMC Plant Biol..

[61] Zobell O., Faigl W., Saedler H., Münster T. (2010). MIKC* MADS-box proteins: Conserved regulators of the gametophytic generation of land plants. Mol. Biol. Evol..

[62] Urbanus S.L., de Folter S., Shchennikova A.V., Kaufmann K., Immink R.G., Angenent G.C. (2009). In planta localisation patterns of MADS domain proteins during floral development in Arabidopsis thaliana. BMC Plant Biol..

[63] Lin Z., Cao D., Damaris R.N., Yang P. (2020). Genome-wide identification of MADS-box gene family in sacred lotus (*Nelumbo nucifera*) identifies a SEPALLATA homolog gene involved in floral development. BMC Plant Biol..

[64] Gabaldón T., Koonin E.V. (2013). Functional and evolutionary implications of gene orthology. Nat. Rev. Genet..

[65] Hurst L.D. (2002). The Ka/Ks ratio: Diagnosing the form of sequence evolution. Trends Genet..

[66] Saha G., Park J.-I., Jung H.-J., Ahmed N.U., Kayum M.A., Chung M.-Y., Hur Y., Cho Y.-G., Watanabe M., Nou I.-S. (2015). Genome-wide identification and characterization of MADS-box family genes related to organ development and stress resistance in *Brassica rapa*. BMC Genom..

